# Supplementary material for: Vitrification and nanowarming enable long-term organ cryopreservation and life-sustaining kidney transplantation in a rat model
Source: Nat Commun. 2023 Jun 9;14:3407. doi: 10.1038/s41467-023-38824-8 (PMC10256770; doi:10.1038/s41467-023-38824-8)
Supplement: Supplementary file 1 — Supplementary Information [file 41467_2023_38824_MOESM1_ESM.pdf]

Supplementary Information

Title

Vitrification and nanowarming enable long-term organ cryopreservation and life-sustaining kidney transplantation in a rat model

Author list

Zonghu Han[1, †], Joseph Sushil Rao[2, 3, †], Lakshya Gangwar[1], Bat-Erdene Namsrai[2], Jacqueline L. Pasek-Allen[1, 4], Michael L. Etheridge[1], Susan M. Wolf[5], Timothy L. Pruett[2], John C. Bischof[1, 6, *], and Erik B. Finger[2, *]

Author notes:

†, These authors contributed equally: Zonghu Han and Joseph Sushil Rao.
*, These authors jointly supervised this work: John C. Bischof, and Erik B. Finger.

Affiliations

[1]Department of Mechanical Engineering, University of Minnesota; Minneapolis, MN, USA

[2]Department of Surgery, University of Minnesota; Minneapolis, MN, USA

[3]Schulze Diabetes Institute, University of Minnesota; Minneapolis, MN, USA

[4]Department of Biomedical Engineering, University of Minnesota; Minneapolis, MN, USA

[5]Consortium on Law and Values in Health, Environment & the Life Sciences, University of Minnesota, Minneapolis, MN, USA

[6]Institute for Engineering in Medicine, University of Minnesota; Minneapolis, MN, USA

Corresponding authors

Correspondence to Erik B. Finger (efinger@umn.edu) or John C. Bischof (bischof@umn.edu)

Table of Contents

Supplementary results 3

CPA and perfusion protocol optimization 3

Microscopy of control, CPA perfused, and nanowarmed kidneys 4

Nanowarmed allogeneic kidney transplant 5

Supplementary Discussion 5

Design of CPA loading and unloading protocols 5

Exclusion criteria based on perfusion performance 7

Iron oxide nanoparticles 7

Additional predictors of long-term function 7

Supplementary Figures 9

Supplementary Fig. 1. Comparison between previously published and the modified loading protocol of VMP 9

Supplementary Fig. 2. Comparison between frozen and vitrified samples by micro-CT and differential scanning calorimetry 10

Supplementary Fig. 3. CPA equilibration during perfusion loading and removal 11

Supplementary Fig. 4. Expanded histologic appearance of nanowarmed rat kidneys and control groups 14

Supplementary References 14

Supplementary results

CPA and perfusion protocol optimization

We previously tested rat kidney vitrification and nanowarming using stepwise loading of a well-known CPA (VS55) at a constant flow rate [1]. Under those conditions, we could achieve ice-free vitrification and rewarm organs without ice formation or cracking. However, the histologic appearance suggested that we did not achieve full biologic recovery. The results indicated that CPA-induced injury, not vitrification and nanowarming, was the limiting factor for success. This observation led us to look for a less toxic CPA and improved loading protocol.

We elected to explore the CPA VMP, which has a similar concentration to VS55 (8.4 M). We initially started with the loading protocol developed by Fahy et al. in rabbit kidneys [2-4]. The protocol was originally developed as an intermediary step before loading an even more concentrated (9.3M) CPA called M22. The protocol included an initial flush of the rat kidney with hypothermic CPA carrier solution (LM5) at 4 °C, a ramp from 0 to 5 M VMP as the temperature was gradually decreased from 4 °C to -3 °C, then 10 minutes of perfusion at 5 M VMP at -3 °C. Finally, the VMP was increased to 8.4 M (full strength) as the temperature dropped to -22 °C for 20 minutes (Supplementary Fig. 1a). We elected to stop at VMP rather than proceed to full-strength M22 to avoid dropping to -22 °C to minimize toxicity. After CPA loading, the rat kidneys were cooled to -150 °C using a controlled rate freezer. Visually, the solution surrounding the kidneys (8.4 M VMP) appeared glassy (i.e., no ice) (Supplementary Fig. 1e). However, the kidneys appeared frozen by micro-CT, with a radiodensity consistent with ice (yellow/green in the pseudocolor scale) rather than the (red/orange) of the surrounding CPA (Supplementary Fig. 1f and discussed further below). The decreased radiodensity of the kidney suggested ice formation had occurred and vitrification had not been achieved [1,5].

We speculated that these kidneys did not appear vitrified because the original VMP loading protocol did not deliver sufficient CPA to the tissue. To test this possibility and to develop alternative loading strategies to overcome it, we developed a mass transport model based on a Krogh cylinder where the kidney is represented by many identical cylindrical/hexagonal prism units in parallel, each of which consists of a central capillary surrounded by tissue. The mass transfer in the whole kidney was analyzed by considering each cylinder as a functional unit representative of the processes occurring in parallel throughout the whole organ.

Both CPA and water transport (solute and solvent flow) occur between the capillary blood vessel, fed by the perfusate, and the surrounding cells (i.e., parenchyma). Each needs to be suitably loaded with CPA to avoid ice formation. Coupled CPA and water transport between the vascular and cellular compartments of the Krogh cylinder occurred through a composite membrane (basement and cell membrane) and was assumed to be governed by irreversible thermodynamics based on Kedem-Katchalsky (KK) equations [6]. Our model predicted that the tissue VMP concentration using the published protocol only achieved 7.0 M (Supplementary Fig. 1a), which was insufficient to vitrify with the cooling rates we observed, thus explaining the appearance of ice in the kidneys.

Using both the model and empiric testing to guide optimization, we modified the perfusion protocol to improve CPA delivery as follows: 1) Adding impermeant CPA compounds (X-1000 and Z-1000) to the carrier solution to improve tissue dehydration, thereby increasing the vascular diameter, decreasing vascular resistance, and increasing flow to aid loading. 2) Increasing perfusion pressure from 40 mmHg to 60 mmHg for the final step of CPA loading, which increases the flow rate and bulk CPA delivery to the capillary bed, thereby maintaining capillary CPA concentration closer to the inflow concentration and driving CPA transport across the membrane into the surrounding cellular structures (i.e., parenchyma). 3) Maintaining temperature at 4 °C rather than reducing it to -22 °C during perfusion, which decreases viscosity and increases transport, both of which increase the overall delivery of CPA to the tissue. The model predicted that the improved protocol would achieve a tissue CPA concentration of 7.8 M (Supplementary Fig. 1b), sufficient for vitrification as seen visually and by micro-CT (Supplementary Fig. 1g, h).

Further examination of the difference between the original VMP loading protocol that achieved 7.0 M (83% of full strength) in the tissue (and failed vitrification) and the modified protocol that reached 7.8 M (93% of full strength and was successfully vitrified) was performed using micro-CT. Samples of VMP at a range of concentrations were cooled to -150 °C and imaged. From 0 M to ~7.5 M VMP, a direct linear relationship existed between CPA concentration and X-ray attenuation (in HU). At full strength VMP (8.4 M), the HU values were offset from the line established by the lower concentration values, confirming our previous observations that vitrified substances had a higher radiodensity than frozen ones at the same CPA concentration [1,5]. Using this imaging approach, we found that at ≤ 91% of full strength (7.6 M), the VMP samples froze under the cooling conditions; at ≥ 93% (7.8 M), samples were successfully vitrified; in between, there was a transitional zone (Supplemental Fig. 2f). We similarly measured the ice fraction formed during cooling of those concentrations of VMP using differential scanning calorimetry (Supplemental Fig. 2g). Very little ice (ice fraction < 0.1%) was formed above 92% VMP (7.7 M), confirming the micro-CT findings. By micro-CT, we estimated that the modified VMP loading protocol achieved 7.9 M (94%) in the renal parenchyma.

Finally, to further confirm the adequacy of VMP loading, we measured the CPA concentration in the venous and ureter effluents by refractometry (Supplemental Fig. 3) using the modified loading protocol. Relative to full-strength CPA, the VMP concentration at the end of loading was 98.8 ± 0.1% in the venous effluent and 97.4 ± 0.9 % in the ureter. The concentration in the ureter likely reflects the medullary loading, and the vein reflects the entire kidney. Based on those values, we would expect all areas of the kidney would vitrify with the modified CPA loading protocol.

Microscopy of control, CPA perfused, and nanowarmed kidneys

Larger format histologic appearance of nanowarmed and control group kidneys are shown in Supplemental Data Fig. 4. Following is the full histologic interpretation of kidneys for relevant treatment groups:

Fresh control and VMP-only: No histological differences. Glomerulus, Bowman's space, proximal convoluted tubules, distal convoluted tubules, and collecting ducts were normal. Blood vessels were normal, and basement membranes were intact.

VS55-only: Increased Bowman's space with normal blood vessels, diffuse tubular necrosis and hyaline change with no vascular compromise. Diffuse necrosis in collecting ducts.

24-hour UW cold storage: Mild mesangial hypercellularity and focal proximal convoluted tubule necrosis. No glomerular or collecting duct changes.

Fresh control transplants at posttransplant day 30: Focal tubular necrosis in some areas with some hyaline change. Otherwise, normal histology.

Vitrified and nanowarmed kidneys at posttransplant day 30: Focal tubular necrosis and hyaline change with no vascular compromise and intact basement membrane in all nanowarmed and transplanted kidneys. Findings suggest definitive recovery. Yellowish brown material (Marked with black arrows) in some thin-walled capillaries, best seen at the cortico-medullary junction and medulla and absent in glomeruli. There was no apparent cellular reaction to it and no vascular compromise. This may represent residual nanoparticles due to their appearance with polarized light. This is consistent with prior work in rat kidney perfusion and wash-out with IONPs that has suggested that residual IONP amounts remain below tolerable limits [1,7], which is supported here by the post-transplant results.

60-hour UW cold storage, transplant, and immediate explant: Mesangial hypercellularity, diffuse proximal convoluted tubule necrosis with glomerular congestion. Collecting ducts were normal. Larger blood vessels were normal.

Nanowarmed allogeneic kidney transplant

We also examined a limited number (n = 2) of transplants using an outbred rat strain (Sprague Dawley to Sprague Dawley). In this minimal dataset, there was a similar creatinine trajectory between control kidney transplants and nanowarmed kidney transplants, as seen in Lewis rats. Nanowarmed kidney recipient creatinine peaked at 12.2 mg/dL on day 3 and returned to < 2.0 mg/dL by day 20, stabilizing between 1.3-1.6 mg/dL to the end of follow-up.

Supplementary Discussion

Design of CPA loading and unloading protocols

At the outset of this study, we sought to develop a CPA loading and unloading approach to overcome our prior limitations attributed to CPA injury [1]. To achieve this, we focused on reducing chemical toxicity from the CPA, osmotic injury from cell shrinking and swelling during exposure to high osmolality CPA solutions and subsequent washout, and physical injury (i.e., temperature and barotrauma) from perfusion.

First, we addressed chemical toxicity by switching from our prior CPA (VS55) to VMP. Although VS55 had been a commonly used CPA cocktail, the extensive study of CPA toxicity by Fahy and colleagues demonstrated that replacing the propylene glycol in VS55 with ethylene glycol significantly reduced toxicity [2,4]. With this modification, they developed a family of CPA cocktails that showed adequate glass-forming ability with reduced toxicity profiles. Of these, we selected VMP, which has a much better toxicity profile than VS55 and had an acceptable CCR (2 °C/min) and CWR (50 °C/min) in tissue.

Second, we considered osmotic injury. CPAs contain cell-permeant compounds (i.e., DMSO) and cell-impermeant ones (i.e., sugars). Cells shrink due to an efflux of water when exposed to hypertonic solutions of either permeant or impermeant agents. However, as permeant agents gradually equilibrate across the cell membrane, water follows, and the cells return to their original size. For impermeant agents, no equilibration occurs, and cell volume remains shrunken. In our prior work [1], we used step increases in CPA concentration during loading to allow periods of equilibration and to minimize the extent of volumetric cell excursion. This study extended this concept to a continuous ramp increase of cell-permeant CPA compounds to reduce osmotic shifting, at least during the initial loading phases. Decisions regarding the ramp rate (slope of increase in CPA concentration) required balancing a minimization of volumetric cell change, which favors a longer loading time, and CPA toxicity, which favors shorter exposure. Our initial ramp loading protocol was based on that developed by Fahy et al. [4]. However, we later used both experimental and modeling efforts with the Krogh cylinder approach to further optimize loading and unloading.

Third, we considered the system temperature during loading and unloading, which can also drive opposing outcomes. For instance, higher temperature favors improved transport across the Krogh cylinder membrane from the vasculature to parenchyma (and therefore quicker loading), but lower temperature reduces toxicity. Since VMP is relatively nontoxic to renal tissues at 0-4 °C and chilling injury is minimal under those conditions [4], we selected 4 °C for our study. Decreasing temperature from 4 °C to -22 °C decreases diffusivity to 47.5% of the original value [8].

Fourth, we considered perfusion pressure, which balances improved bulk CPA delivery to the capillary beds versus the risk of barotrauma. In Fahy's work and in our mass transport Krogh modeling, 40 mmHg was insufficient to maintain adequate flow rates when the viscosity rose with the increase in CPA concentration to full strength (8.4 M). Typical perfusion pressures in kidneys are sub-physiologic (30-40 mmHg) when organs are perfused with acellular solutions. We kept perfusion at 40 mmHg for the initial loading, but when the flow rate began to drop at high CPA concentration, we increased it to 60 mmHg, which is still lower than used for even higher concentration CPAs such as 9.3 M M22, which is perfused at 80 mmHg [3].

Each of these considerations was incorporated in our Krogh cylinder mass transfer model, which predicted that the original loading protocol reported for VMP [4] would result in insufficient tissue CPA concentration to prevent ice formation during cooling, a finding we verified experimentally. By preloading our system with impermeant CPAs X-1000 and Z-1000 to increase flow rate by tissue dehydration, maintaining the temperature at 4 °C to improve diffusion, and increasing the pressure and duration of full-strength VMP perfusion, we were able to increase the

tissue CPA concentration enough to both vitrify and successfully rewarm kidneys. Further, the switch from VS55 to VMP, with the additional loading condition modifications, reduced toxicity sufficiently to recover transplantable organs that fully restored renal function.

Exclusion criteria based on perfusion performance

Based on our preliminary data (unpublished and [1]), we noted that in some experiments, the IONPs appeared to have aggregated prior to loading. The perfusion resistances were elevated, and the visual appearance of bisected kidneys following unloading showed an accumulation of the dark IONPs in the medullary rays and at the cortical-medullary junction. The cortex was visually free of IONPs, but small areas of aggregates were occasionally noted histologically. While those IONP preparations were stable in CPA solutions as measured by dynamic light scattering (DLS), we assume that a fraction of the IONPs were aggregating, or changing otherwise, resulting in accumulation in the long and thin microvasculature of the renal medulla during perfusion flow.

To overcome the possible aggregation, we filtered IONPs before use (with progressively smaller mesh pore sizes down to 2 μm). That change led to improved perfusion loading (lower pressure) and a lack of visible kidney accumulation after washout. All experiments in this study used pre-perfusion filtering. However, to avoid misinterpreting experiments where aggregation (or other technical problems such as vascular kinking or air bubble entrapment) might occur, we established a prospective exclusion criterion that the flow rate during unloading needed to be > 45% of the flow rate during loading and needed to stay at that level or increase as CPA was removed in order to be included in this study. Only a single kidney failed this exclusion test, and that one had not had the IONP filtered on the day of use, as was our standard protocol.

Iron oxide nanoparticles

Another critical element in achieving nanowarming success was the synthesis of nanoparticles that are biocompatible, colloidally stable in CPA, high heating, and can be washed out of the organ during unloading. We used silica- and PEG-coated IONPs, which achieve these performance metrics [7]. Importantly, these IONPs remained within the vasculature and were not taken up by cells at low temperatures (< 4 °C), where endocytosis is minimal. We have shown that the small residual iron content from retained IONPs was below FDA-approved equivalent dosing for intravenous iron therapy and is nontoxic at those levels in vivo [7,9,10]. We found no evidence of systemic toxicity in transplant recipients apart from a minor elevation of amylase, which is relatively non-specific and is of uncertain significance. The small amount of yellowish-brown material in the thin-walled capillaries that were occasionally visible in histology may have been small quantities of retained IONPs, but this remains to be clarified. The perfused IONP concentration was sufficient to rewarm kidneys fast enough to avoid significant ice crystallization during rewarming. However, higher concentrations of particles (i.e., more iron content/mL) [11] or increased RF magnetic field strength (and/or frequency) have been shown to increase warming rates up to 100s °C/min, thereby potentially allowing a further reduction in the required CPA concentration.

Additional predictors of long-term function

We can place the kidney function on a clinical scale of organ recovery, which includes immediate graft function (rapid decline in creatinine without dialysis), slow graft function (recipient creatinine > 3.0 mg/dL on postop day 5 but not needing dialysis [12]), and delayed graft function (need for dialysis in the first week following transplant [13]). Nanowarmed kidney recipients produced urine intraoperatively and did not require dialysis or pharmacologic correction of renal dysfunction and electrolyte abnormalities. By these criteria, nanowarmed kidneys demonstrated slow graft function. While slow graft function is a risk for developing acute rejection in clinical transplantation, if that rejection can be avoided, these kidneys have similar outcomes as those with immediate graft function [12] and much better than those with delayed graft function [14].

Supplementary Figures

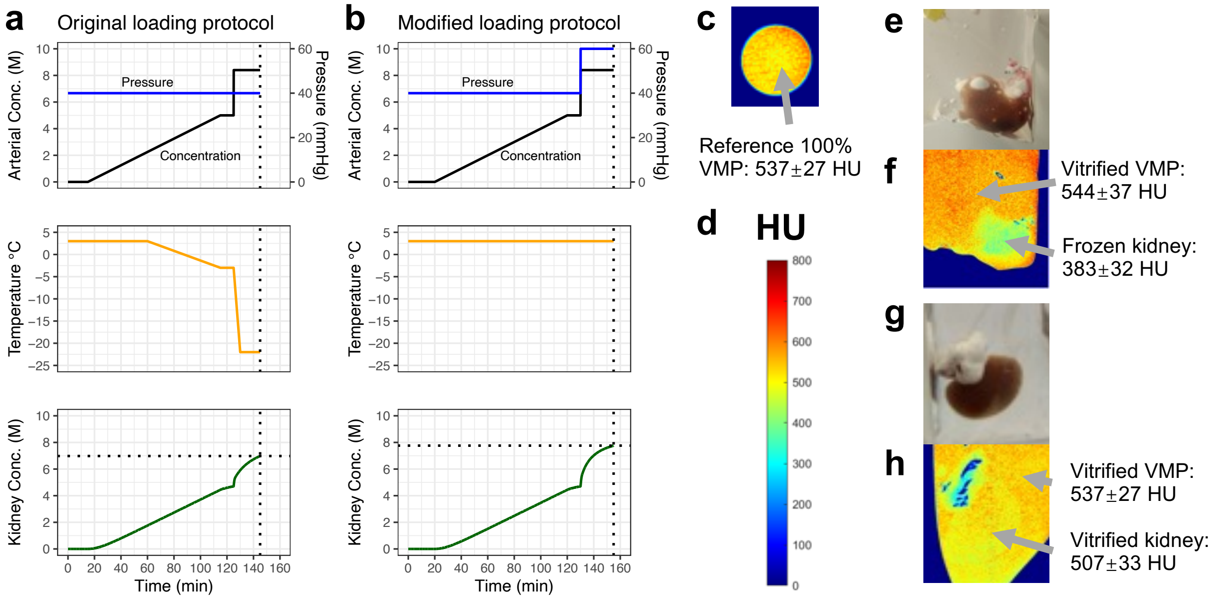

Supplementary Fig. 1. Comparison between previously published and the modified loading protocol of VMP. a, CPA concentration, perfusion pressure and temperature, and the Krogh cylinder model-predicted CPA concentration inside the kidney parenchyma during the loading using the previous protocol. **b,** CPA concentration, perfusion pressure and temperature, and the Krogh cylinder model-predicted CPA concentration inside the kidney parenchyma during the loading using the modified protocol. **c-d,** Representative micro-CT image of vitrified CPA solution and pseudocolor scale of radiodensity in HU. **e-f,** Representative photograph (**e**) and pseudocolor image acquired by micro-CT (**f**) of the frozen VMP-loaded kidney using the previous protocol. **g-h**, Representative photograph (**g**) and pseudocolor image acquired by micro-CT (**h**) of the vitrified VMP-loaded kidney using the modified protocol. HU, Hounsfield units.

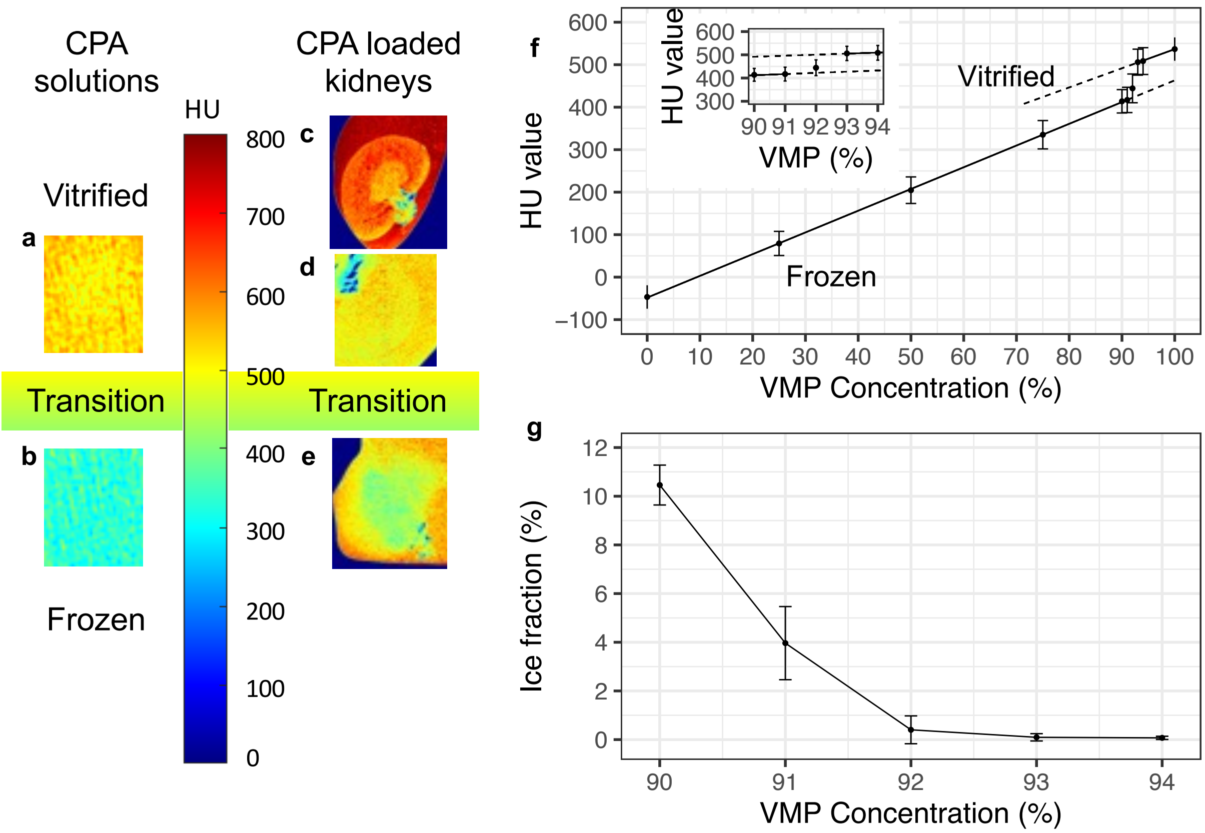

Supplementary Fig. 2. Comparison between frozen and vitrified samples by micro-CT and differential scanning calorimetry. **a-e,** Micro-CT pseudocolor images of (**a**) vitrified VMP, (**b**) frozen VMP, (**c**) vitrified kidney loaded with CPA and IONP, (**d**) vitrified kidney, and (**e**) frozen kidney. **f**, Radiodensity in HU acquired by micro-CT for diluted VMP samples at concentrations of 0 to 100% of full strength (8.4 M). Two parallel regression lines show frozen (lower) and vitrified (upper) conditions. The difference between lines is the offset for vitrified samples which is used to show success in vitrification. **g,** Ice fraction (%) of the diluted VMP samples (90, 91, 92, 93, and 94%) acquired by differential scanning calorimetry at the cooling rate of 10 °C/min. Data are mean ± s.d. $n = 3$.   HU, Hounsfield units; IONP, iron oxide nanoparticles.

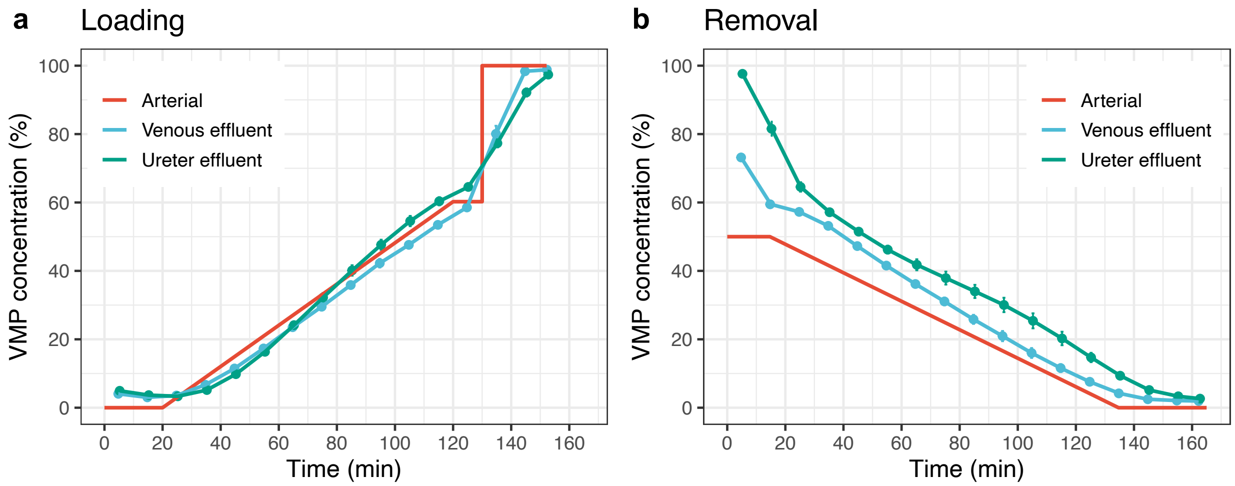

Supplementary Fig. 3. CPA equilibration during perfusion loading and removal. a, Venous and ureteral effluent CPA (VMP) concentration determined by refractometry during perfusion loading and compared to the arterial perfusate concentration. **b,** Unloading/removal of VMP with the same measurements. Data are mean ± s.d. n = 3 independent experiments for venous and ureteral sampling.

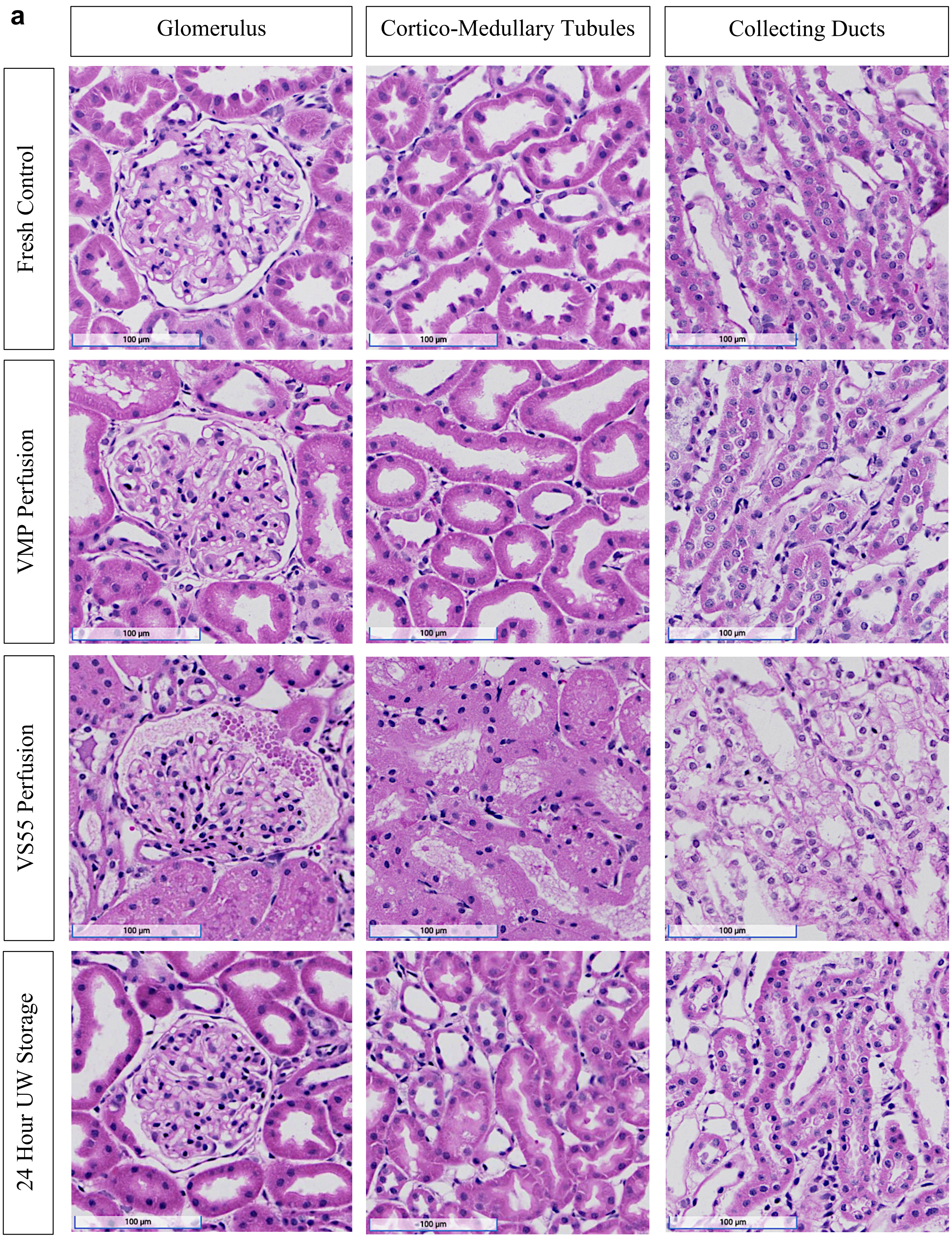
a
Glomerulus
Cortico-Medullary Tubules
Collecting Ducts
Fresh Control
VMP Perfusion
VS55 Perfusion
24 Hour UW Storage
100 μm

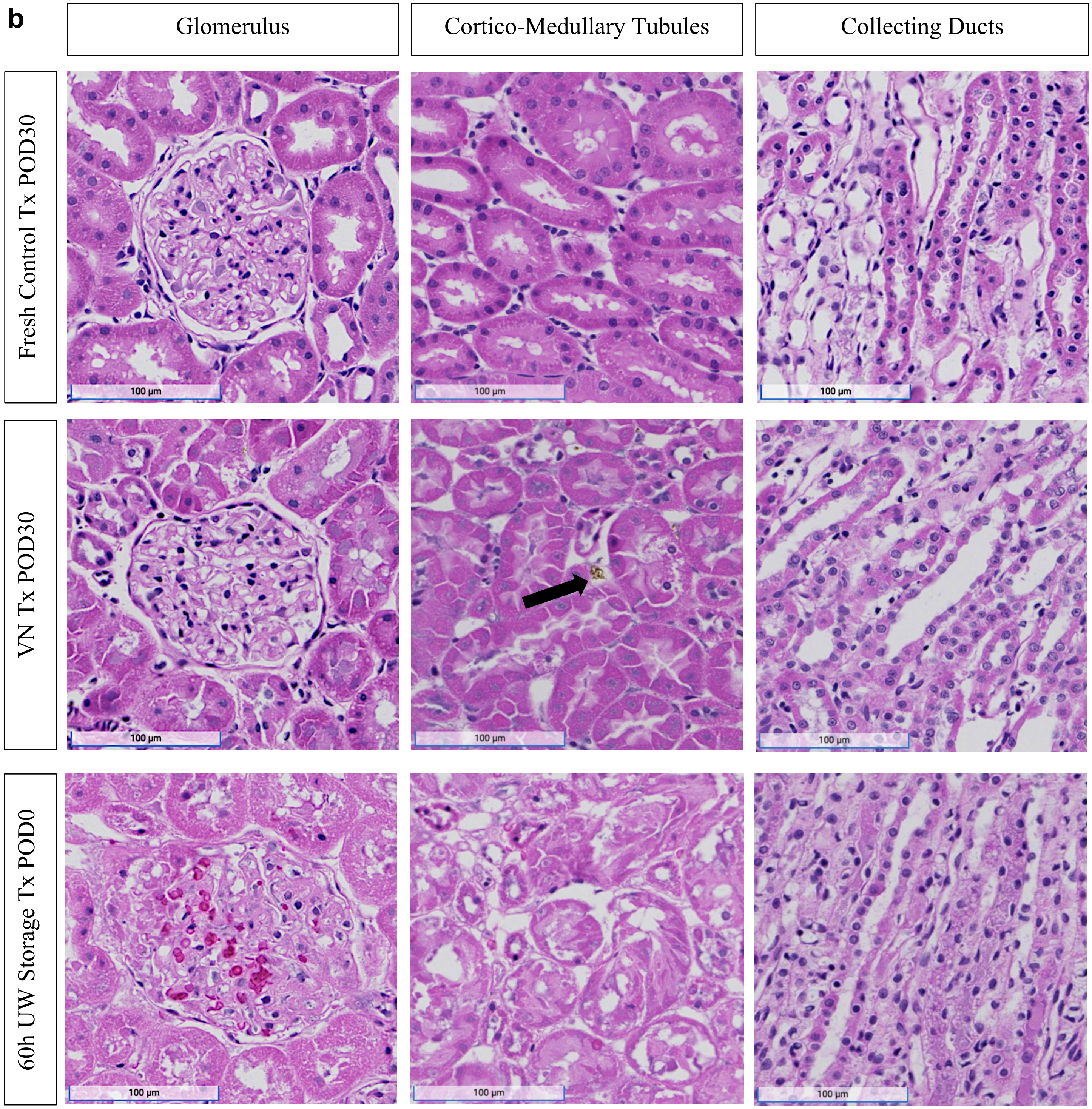
b
Glomerulus
Cortico-Medullary Tubules
Collecting Ducts
Fresh Control Tx POD30
VN Tx POD30
60h UW Storage Tx POD0
100 µm

Supplementary Fig. 4. Expanded histologic appearance of nanowarmed rat kidneys and control groups. a, Histological changes (H&E) of renal glomeruli, cortico-medullary tubules, and collecting ducts of fresh control, CPA-only (VS55-only and VMP-only) treated organs, and 24-hour cold storage in UW solution. **b,** Histologic appearance of fresh control and nanowarmed kidney transplants at day 30 posttransplant and 60-hour cold stored in UW kidney immediately following transplant. Black arrow shows presumed residual nanoparticles. Scale bars are 100 µm. CPA, cryoprotective agent; H&E, hematoxylin and eosin; POD, post operative day; Tx, transplant; VN, vitrified and nanowarmed.

Supplementary References

1. Sharma, A., *et al.* Vitrification and Nanowarming of Kidneys. *Adv Sci (Weinh)* **8**, e2101691 (2021).
2. Fahy, G.M., Wowk, B., Wu, J. & Paynter, S. Improved vitrification solutions based on the predictability of vitrification solution toxicity. *Cryobiology* **48**, 22-35 (2004).
3. Fahy, G.M., *et al.* Physical and biological aspects of renal vitrification. *Organogenesis* **5**, 167-175 (2009).
4. Fahy, G.M., *et al.* Cryopreservation of organs by vitrification: perspectives and recent advances. *Cryobiology* **48**, 157-178 (2004).
5. Bischof, J.C., Mahr, B., Choi, J.H., Behling, M. & Mewes, D. Use of X-ray tomography to map crystalline and amorphous phases in frozen biomaterials. *Ann Biomed Eng* **35**, 292-304 (2007).
6. Kedem, O. & Katchalsky, A. Thermodynamic analysis of the permeability of biological membranes to non-electrolytes. *Biochim Biophys Acta* **27**, 229-246 (1958).
7. Gao, Z., *et al.* Preparation of Scalable Silica-Coated Iron Oxide Nanoparticles for Nanowarming. *Advanced Science* **7**, 1901624 (2020).
8. Jomha, N.M., *et al.* Permeation of several cryoprotectant agents into porcine articular cartilage. *Cryobiology* **58**, 110-114 (2009).
9. Zhang, J., *et al.* Quantification and biodistribution of iron oxide nanoparticles in the primary clearance organs of mice using T1 contrast for heating. *Magn Reson Med* **78**, 702-712 (2017).
10. Ring, H.L., Bischof, J.C. & Garwood, M. *The use and safety of iron-oxide nanoparticles in MRI and MFH*, (eMagRes, 2021).
11. Pasek-Allen, J.L., Wilharm, R.K., Gao, Z., Pierre, V.C. & Bischof, J.C. Phosphonate coating of commercial iron oxide nanoparticles for nanowarming cryopreserved samples. *J Mater Chem B* **10**, 3734-3746 (2022).
12. Humar, A., *et al.* Effect of initial slow graft function on renal allograft rejection and survival. *Clin Transplant* **11**, 623-627 (1997).
13. Yarlagadda, S.G., *et al.* Marked variation in the definition and diagnosis of delayed graft function: a systematic review. *Nephrol Dial Transplant* **23**, 2995-3003 (2008).
14. Zeraati, A.A., Naghibi, M., Kianoush, S. & Ashraf, H. Impact of slow and delayed graft function on kidney graft survival between various subgroups among renal transplant patients. *Transplant Proc* **41**, 2777-2780 (2009).
