# Supplementary material for: Vitrification and nanowarming enable long-term organ cryopreservation and life-sustaining kidney transplantation in a rat model
Source: Nat Commun. 2023 Jun 9;14:3407. doi: 10.1038/s41467-023-38824-8 (PMC10256770; doi:10.1038/s41467-023-38824-8)
Supplement: Supplementary file 3 — Description of Additional Supplementary Files [file 41467_2023_38824_MOESM3_ESM.pdf]

<u>Description of Additional Supplementary Files Document</u>

Supplementary Data 1- Summary of statistical treatment for each figure.
