# Supplementary material for: Vitrification and nanowarming enable long-term organ cryopreservation and life-sustaining kidney transplantation in a rat model
Source: Nat Commun. 2023 Jun 9;14:3407. doi: 10.1038/s41467-023-38824-8 (PMC10256770; doi:10.1038/s41467-023-38824-8)
Supplement: Supplementary file 5 — Reporting Summary [file 41467_2023_38824_MOESM5_ESM.pdf]

nature portfolio

Corresponding author(s): John C. Bischof and Erik B. Finger

Last updated by author(s): 5/12/2023

Reporting Summary

Nature Portfolio wishes to improve the reproducibility of the work that we publish. This form provides structure for consistency and transparency in reporting. For further information on Nature Portfolio policies, see our Editorial Policies and the Editorial Policy Checklist.

Statistics

For all statistical analyses, confirm that the following items are present in the figure legend, table legend, main text, or Methods section.

n/a	Confirmed	
☐	☒	The exact sample size (*n*) for each experimental group/condition, given as a discrete number and unit of measurement
☐	☒	A statement on whether measurements were taken from distinct samples or whether the same sample was measured repeatedly
☐	☒	The statistical test(s) used AND whether they are one- or two-sided<br>*Only common tests should be described solely by name; describe more complex techniques in the Methods section.*
☒	☐	A description of all covariates tested
☐	☒	A description of any assumptions or corrections, such as tests of normality and adjustment for multiple comparisons
☐	☒	A full description of the statistical parameters including central tendency (e.g. means) or other basic estimates (e.g. regression coefficient) AND variation (e.g. standard deviation) or associated estimates of uncertainty (e.g. confidence intervals)
☐	☒	For null hypothesis testing, the test statistic (e.g. *F*, *t*, *r*) with confidence intervals, effect sizes, degrees of freedom and *P* value noted<br>*Give P values as exact values whenever suitable.*
☒	☐	For Bayesian analysis, information on the choice of priors and Markov chain Monte Carlo settings
☒	☐	For hierarchical and complex designs, identification of the appropriate level for tests and full reporting of outcomes
☐	☒	Estimates of effect sizes (e.g. Cohen's *d*, Pearson's *r*), indicating how they were calculated

Our web collection on statistics for biologists contains articles on many of the points above.

Software and code

Policy information about availability of computer code

Data collection	*Provide a description of all commercial, open source and custom code used to collect the data in this study, specifying the version used OR state that no software was used.*
Data analysis	The following softwares were used for data analysis: R 4.2.1, MATLAB 2019a, 3D CT pro (Nikon Metrology, MI), VGstudio Max 3.2 (Volume Graphics, NC),

For manuscripts utilizing custom algorithms or software that are central to the research but not yet described in published literature, software must be made available to editors and reviewers. We strongly encourage code deposition in a community repository (e.g. GitHub). See the Nature Portfolio guidelines for submitting code & software for further information.

Data

Policy information about availability of data

All manuscripts must include a data availability statement. This statement should provide the following information, where applicable:

- Accession codes, unique identifiers, or web links for publicly available datasets
- A description of any restrictions on data availability
- For clinical datasets or third party data, please ensure that the statement adheres to our policy

The raw data for each figure is available in the Supplementary information.

Human research participants

Policy information about studies involving human research participants and Sex and Gender in Research.

Reporting on sex and gender	N/A
Population characteristics	N/A
Recruitment	N/A
Ethics oversight	N/A

Note that full information on the approval of the study protocol must also be provided in the manuscript.

Field-specific reporting

Please select the one below that is the best fit for your research. If you are not sure, read the appropriate sections before making your selection.

☒ Life sciences ☐ Behavioural & social sciences ☐ Ecological, evolutionary & environmental sciences

For a reference copy of the document with all sections, see nature.com/documents/nr-reporting-summary-flat.pdf

Life sciences study design

All studies must disclose on these points even when the disclosure is negative.

Sample size	We first examined preliminary results on urine output (over 40 min) during normothermic machine perfusion and compared two cryoprotective agents (VS55 and VMP). If we use mean A = 1.11, mean B = 0.35, sd = 0.3. Using standard assumptions (alpha = 0.05, beta = 0.2, equal distribution between groups), we use the T statistic and determined the minimum sample size in each group (n = 3). However, since not all test parameters examined were normally distributed, we would need a group size of $n \geq 4$ to be able to compare continuous variables with the possibility of demonstrating difference in the mean at a probability $p < 0.05$ for non-parametric tests. Thus, minimum sample size for statistical comparisons in this study were ≥ 3 for normally distributed parameters and ≥ 4 for non-normal.
Data exclusions	We excluded any kidneys that had a unloading flow rate < 45% of the flow during loading. We did so to avoid misinterpreting technical problems from catheter or vascular kinking, air bubble entrapment, or nanoparticle aggregation.
Replication	Experiments were reproduced on separate days over several months using different batches of primary reagents (silica coated iron oxide nanoparticles and cryoprotective agent solutions). Each individual data point represents a unique donor kidney (and unique recipient rat for transplant experiments). After working out the technical aspects of the protocol such as nanoparticle synthesis and filtration, cannulation approach for rats, and transplant technique, there were no failures. Early in preliminary experiments we saw failure from ice formation that was overcome by adjusting the CPA perfusion protocol (detailed in the supplemental materials), but after the adjustments described in the paper we saw no further failures. Also, in preliminary experiments we sacrificed one rat on day 3 after transplant of a nanowarmed kidney due to hyporventilation/hypercarbia from too high of a dose of long acting pain medication (buprenorphine SR) at the advice of our veterinary staff. That animal had a functioning transplant at sacrifice. The surgical technique, anesthetics, and pain medications optimized in a revised protocol and all the experiments here were from the revised protocol. The total replicates for each experiment is shown in the figure legends, attached full dataset, and statistical summaries. Generally there were 4 independent experiments for each group in the normothermic perfusion assays and 5-6 independent experiments for each transplant group. Once the final protocol was implemented, there were no failures.
Randomization	There was random selection of rats for experiments. Rats were aged in our facility to achieve the target weight (450-525 gm) and the rats were selected at random for the experiments. We rotated between groups (fresh control, CPA-only, nanowarmed, 24 hr-cold stored) for experiments. All rats came from the same vendor, were the same sex, and were housed in the same room.
Blinding	Subjective interpretation of histology was performed by external specialists in blinded fashion.

Reporting for specific materials, systems and methods

We require information from authors about some types of materials, experimental systems and methods used in many studies. Here, indicate whether each material, system or method listed is relevant to your study. If you are not sure if a list item applies to your research, read the appropriate section before selecting a response.

Materials & experimental systems

n/a	Involved in the study
☒	☐ Antibodies
☒	☐ Eukaryotic cell lines
☒	☐ Palaeontology and archaeology
☐	☒ Animals and other organisms
☒	☐ Clinical data
☒	☐ Dual use research of concern

Methods

n/a	Involved in the study
☒	☐ ChIP-seq
☒	☐ Flow cytometry
☒	☐ MRI-based neuroimaging

Animals and other research organisms

Policy information about studies involving animals; ARRIVE guidelines recommended for reporting animal research, and Sex and Gender in Research

Laboratory animals	450 – 525 gm (16-32 week old) male Lewis rats (Strain #004) and Sprague Dawley rats (Strain #400) were purchased from Charles River Laboratories
Wild animals	The study did not include wild animals .
Reporting on sex	Experiments performed using male rats. Male sex was selected to avoid immunoreactivity in transplants of male organs in female recipients (against HY antigens), or the possibility of graft versus host from female donors reacting to male recipients from donor derived passenger leukocytes. Additionally, larger vessel size in male rats makes the experimental model more reliable. We do not expect the findings will be sex-specific.
Field-collected samples	The study did not include animals collected from the field.
Ethics oversight	The Institutional IACUC committee from the University of Minnesota (protocol #2204-39970A) approved all animal studies.

Note that full information on the approval of the study protocol must also be provided in the manuscript.
